# Mapping Genetically Compensatory Pathways from Synthetic Lethal Interactions in Yeast

**DOI:** 10.1371/journal.pone.0001922

**Published:** 2008-04-09

**Authors:** Xiaotu Ma, Aaron M. Tarone, Wenyuan Li

**Affiliations:** Molecular and Computational Biology Program, Department of Biological Sciences, University of Southern California, Los Angeles, California, United States of America; Fred Hutchinson Cancer Research Center, United States of America

## Abstract

**Background:**

Synthetic lethal genetic interaction analysis has been successfully applied to predicting the functions of genes and their pathway identities. In the context of synthetic lethal interaction data alone, the global similarity of synthetic lethal interaction patterns between two genes is used to predict gene function. With physical interaction data, such as protein-protein interactions, the enrichment of physical interactions within subsets of genes and the enrichment of synthetic lethal interactions between those subsets of genes are used as an indication of compensatory pathways.

**Result:**

In this paper, we propose a method of mapping genetically compensatory pathways from synthetic lethal interactions. Our method is designed to discover pairs of gene-sets in which synthetic lethal interactions are depleted among the genes in an individual set and where such gene-set pairs are connected by many synthetic lethal interactions. By its nature, our method could select compensatory pathway pairs that buffer the deleterious effect of the failure of either one, without the need of physical interaction data. By focusing on compensatory pathway pairs where genes in each individual pathway have a highly homogenous cellular function, we show that many cellular functions have genetically compensatory properties.

**Conclusion:**

We conclude that synthetic lethal interaction data are a powerful source to map genetically compensatory pathways, especially in systems lacking physical interaction information, and that the cellular function network contains abundant compensatory properties.

## Introduction

Genetic interaction analysis, in which the combined mutations of two genes exhibit phenotypes significantly different from the single mutation of either one [1], is a powerful tool allowing biologists to investigate the genetic components of an organism [2]. The International Yeast Gene Deletion Consortium constructed a nearly complete collection of gene-deletion mutants for yeast [3], providing an excellent starting point for the study of genetic interactions [4]. Synthetic lethal interaction analysis, in which the deletion of two viable genes makes the organism (yeast) inviable, generated the first large-scale synthetic lethal interaction data set [5]. The BioGRID database [6] contains 9,376 non-redundant yeast synthetic lethal interactions involving 2348 yeast genes (as of August, 2007).

**Figure 1:**
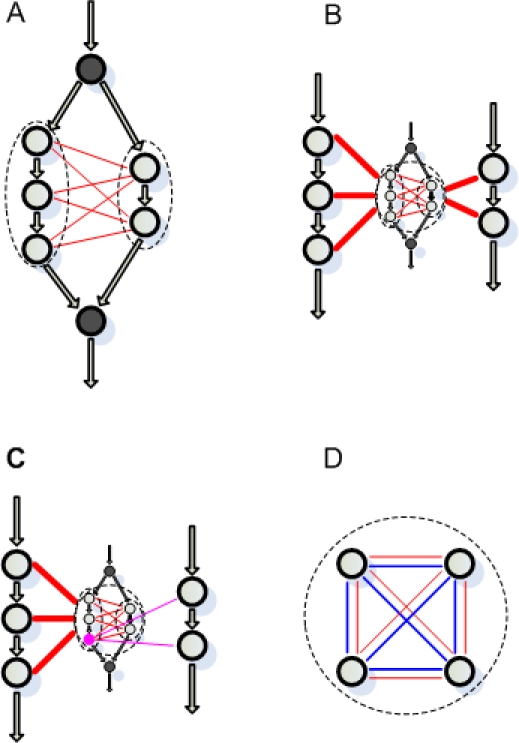
Possible mode of synthetic lethal interactions. Solid circles represent genes, arrows represent information flow, blue lines represent physical interactions and red/pink lines represent synthetic lethal interactions. Dashed circles group genes into pathways. (A–C), Compensatory pathways: (A) The information flow from one master gene (filled circle on the top) can go through either of the two pathways and get to another master gene (filled circle on the bottom). Thus mutation of any single genes in either pathway will not block the information flow, while a double mutation where both pathways are disabled will block the information flow and the cell will die, if this information flow is critical to its survival. However, synthetic lethal interactions are depleted from either pathway since a double deletion in either pathway will not cause lethal effect. (B) Genes involved in the same function generally have similar patterns of synthetic lethal interaction with genes from other pathways. Heavy red lines indicate that all genes grouped by the large dashed circle have synthetic lethal interaction with the other genes. (C) Multifunction effect. A gene (or a pathway) may have multiple functions. All single-function genes (empty circles) grouped by the dashed circle tend to have similar synthetic lethal interaction patterns. However, a multifunction gene, as indicated by the pink circle, may have a synthetic lethal interaction pattern (the additional synthetic lethal interactions shown with pink lines) that differs from the other genes grouped by the dashed circle. (D) Single pathway: A certain protein complex/pathway can sustain the mutation of one, but not two, member genes without loss of function.

All cells must manage biological information to survive. These functions are frequently achieved through various cascades (e.g. physical transport, transcription, translation, phosphorylation, etc.) that involve proteins encoded by different genes in the genome. Due to the involvement of multiple genes in such systems, there is a potential for genetic interactions among members of these pathways and among members of other pathways in the cell that have similar/overlapping functions. Synthetic lethal interactions, where mutations are only lethal in combination, are generally considered to reflect such interactions within and between cascades, with parallel or compensatory pathways explaining most, though not all, synthetic lethal interactions [1], [7]–[8]. This view is illustrated in Figure 1A. Due to the existence of alternative pathways for the information flow, disabling any single gene from either pathway will not block the information flow. However, disabling any two components, one from each pathway, will block the information flow and leads to the death of the cell. Note the depletion of interactions among components from the same pathway, which means that disabling any two components from the same pathway will not block the information flow. Shown in Figure 1B is the fact that components from either of these two pathways often have similar patterns of synthetic lethal interactions with other genes from the organism. Note that an important underlying assumption for Figure 1B is that the genes do not have multifunction, i.e., no genes involved have two or more distinct functions (Figure 1C). Finally, a relatively smaller proportion of synthetic lethal interactions are within pathways that can sustain one, but not two, mutations without loss of function (Figure 1D).

The fact that the compensatory pathway structure can be reflected by synthetic lethal analysis is widely appreciated. For example, Ye et al [7] used a congruence score, which measures global similarity between the patterns of synthetic lethal interactions of two genes, to predict that two genes with high congruence scores were likely in the same pathway and thus share similar functional roles. However, the underlying compensatory structure between pathways (Figure 1A) could not be revealed by this method. Kelley and Ideker [1] proposed a between-pathway model to score the compensatory relationship between pathways in a probabilistic framework. Their method emphasized the enrichment of synthetic lethal interactions connecting two pathways. An equally important component in their work was the enrichment of physical interactions within each pathway, including protein-protein interactions [9]–[10] and protein-DNA interactions [11]. Ulitsky and Shamir [8] extended this idea by requiring that each pathway in a compensatory pair was a connected graph in the physical interaction network rather than enriched with physical interactions. They found that twice as many genetic interaction pairs can be assigned to compensatory pathways. Although the physical interactions within each pathway increased the confidence that the resulting pathways are biologically meaningful, some pathway pairs were sacrificed due to the lack of support of physical interactions, owing to the fact that current physical interaction data contain false positives and false negatives. More importantly, their frameworks did not impose any constraint on synthetic lethal interactions within each pathway. A high-scoring compensatory pathway pair may contain a pathway that has many within-pathway synthetic lethal interactions, which is difficult to explain with the current model (Figure 1A). In addition, in some species there is only genetic screening data available, making it worthwhile to study the power of mapping genetically compensatory pathways using only synthetic lethal interactions.

To account for the above theoretical considerations, we developed a graph theory method to group genes into distinct but compensatory pathway pairs, based solely on the local connectivity structure of the synthetic lethal interaction network. Pathways in each pair were required to be connected by many synthetic lethal interactions and depleted for synthetic lethal interactions within each pathway. A heuristic algorithm was proposed to realize our method. This approach had considerable power in grouping genes into functionally homogenous sets and identified many cellular functions exhibiting genetically compensatory properties. However, we must make it clear that our method was not developed to identify pathways like Figure 1D, which can be identified using the “within-pathway model” of Kelly and Ideker [1].

## Results

### Pathway definition and identification

All genes in one organism interact, forming a gene-network. Given the fact that some genes specifically cooperate to take over some highly specialized cellular functions, pathways are often used to represent part of the gene-network at a more detailed level. An example compensatory pathway pair is shown in Figure 1A, where the three genes shown in left and the two genes shown in right can be defined as two distinct pathways. However, it is also appropriate to group all the five genes (or plus the two master genes) into one pathway. Both definitions make sense because the first definition focuses on the functional redundancy of the two smaller pathways while the second definition focuses on the fact that all these genes are involved in one specific cellular function. Since the synthetic lethal interactions are in general considered to reveal the functional redundancy between pathways [1], [7]–[8], the pathways we pursued in this paper followed the first definition. However, the pathways we identified do not necessarily adhere to this meaning; rather, functionally distinct genes may be found to be compensatory to a multifunction pathway since we did not use the physical interaction data. This fact is discussed further in section “Multifunction effect of pathways”.

**Figure 2:**
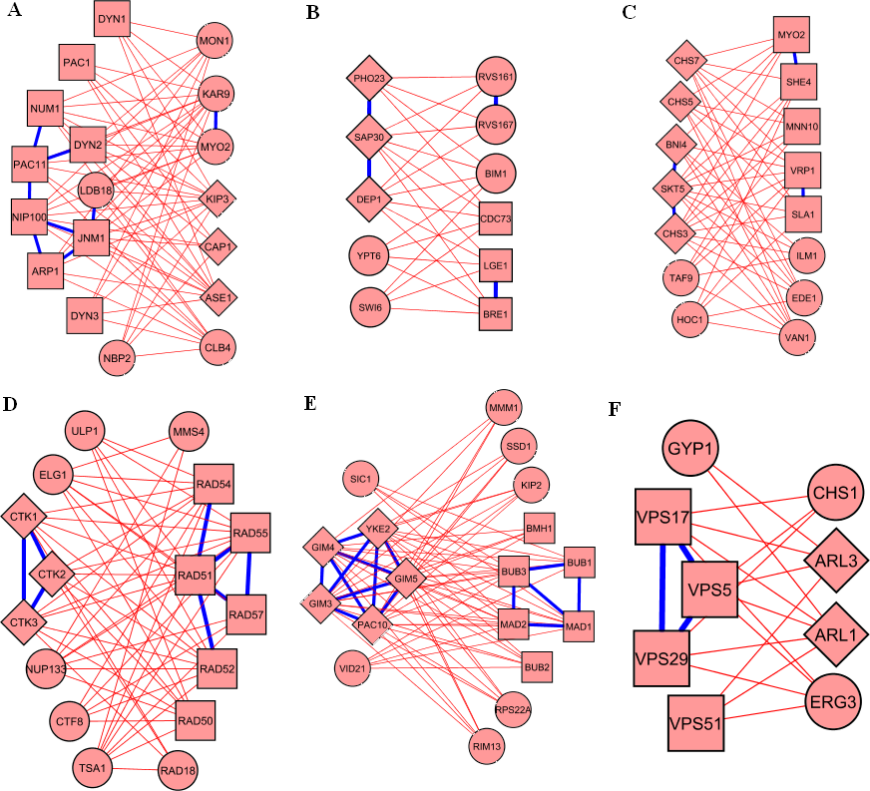
Example pathway pairs identified by our method. Each example is shown in a pair: left: pathway 1, right: pathway 2. Heavy blue lines represent physical interactions (protein-protein interaction and/or protein-DNA interactions) between genes and red lines represent synthetic lethal interactions. Diamonds and Boxes represent genes with the dominant function for pathway 1 and pathway 2, respectively (see text for details). (A) The dynein-dynactin pathway and protein depolymerization. (B) Histone deacetylation and histone methylation. (C) Actin filament-based process and chitin metabolic process. (D) Positive regulation of RNA elongation and double-strand break repair via single-strand annealing. (E) Tubulin folding and spindle checkpoint. (F) Golgi to plasma membrane protein transport and retrograde transport, endosome to Golgi.

Shown in Figure 2 are several genetically redundant pathway pairs identified by our method. For example, the *dynein-dynactin pathway* (shown in boxes, P<1e-12, Hypergeometric test, herein and after, Figure 2A left) and *protein depolymerization pathway* (diamonds, P<1e-6, Figure 2A right) were found to be genetically compensatory. Genetically compensatory pathway pairs also shown in Figure 2 include *histone deacetylation* (diamonds, P<1e-6) and *histone methylation* (boxes, P<1e-6, Figure 2B), *actin filament-based process* (boxes, P<1e-8) and *chitin metabolic process* (diamonds, P<1e-11, Figure 2C), *positive regulation of RNA elongation* (diamonds, P<1e-8) and *double strand break via single strand annealing* (boxes, P<1e-14, Figure 2D), *tubulin folding* (diamonds, P<1e-12) and *spindle checkpoint* (boxes, P<1e-8, Figure 2E) and *Golgi to membrane protein transport* (boxes, P<1e-5) and *endosome to Golgi retrograde transport* (diamonds, P<1e-9, Figure 2F). Known physical interactions that were listed in Ulitsky and Shamir (2005), including protein-protein and protein-DNA interactions, were also indicated in Figure 2 using heavy blue lines.

By removing physical interaction data from the process of identifying genetically compensatory pathways, there was also a greater potential for spurious identifications (discussed later). Though the approach presented here is theoretically sound, predictions should make sense in the face of known physical interactions within and between pathways. Here we have detailed an example to demonstrate the utility of identifying compensatory pathways solely via genetic interaction information.

In Figure 2C, the functions of an actin filament-based process and chitin metabolism were identified as compensatory processes in this analysis. Though these functions seemed superficially disparate based on Gene Ontology [12], the potential mechanism for their interaction became apparent when the loci involved were investigated. The actin filament-based process genes are involved in various aspects of endocytosis, which require the movement of vesicles between the trans-golgi network and the cell membrane via the actin cytoskeleton (www.yeastgenome.org). The movement of vesicles within a cell depends in part on polysaccharide tags attached to the membranes of the structures [13]–[15]. One of these tags is mannan, a polymer of mannose that is important for vesicle transport, cell wall structure, and other functions dependent on protein glycosylation [14].

The genes in the identified actin filament-based process pathway encode proteins for actin/myosin binding that are known to affect secretion polarity within the cell, as well as a mannan polymerase (VAN1) and a mannosyltransferase (MNN10) [16]. These mannose-processing proteins have been shown to be part of golgi-bound complexes composed of either VAN1/MNN9, or MNN10/ANP1/HOC1/MNN9/MNN11, both of which are involved in protein mannosylation [14]. This information provides a connection to the compensatory pathway involved in chitin synthesis, as HOC1 also bears a physical similarity to several other seemingly unrelated glycosyltransferases MNN1, OCH1, SUR1, and CHS1 (a chitin synthase) proteins, indicating the presence of necessary functional components [17] in proteins of both pathways, and revealing the possibility of redundant functions performed by those proteins. Since chitin is a polymer of N-acetylglucosamine, which bears structural similarity to mannose, and is also a critical component of the cell wall, it is not surprising that a protein or complex could be involved in the transport or polymerization of both molecules. N-acetylglucosamine is used to link mannan to yeast proteins [14]–[15], indicating the necessity of the mannan producing complex to recognize and bind sugars to the molecule that is the building block for chitin. It is also possible that the molecular outputs of both complexes (mannan and chitin) are compensatory. Indeed, evidence of overlap between chitin and mannan glycosylation exists as mutations in the MNN10 complex alter chitin levels in yeast cells [16], [18]. This indicates that either the complexes themselves are partially redundant, or that cell wall stress resulting from missing cell wall mannan can be relieved by the increased production of chitin. Given these similarities in sugar and protein structures and functions between mannan and chitin-based biological processes, it was not surprising (and expected) that they were identified as compensatory processes in this analysis.

### Summary statistics of compensatory pathway pairs

**Figure 3:**
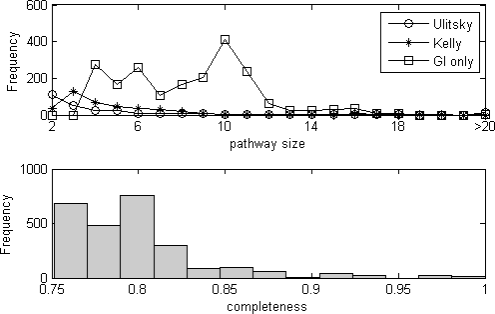
Summary statistics of the pathway pairs we identified. Upper panel: Distribution of the sizes of the pathways of our analysis (GI only; box) and that of Ulitsky and Shamir ([8]; circle) and Kelley and Ideker ([1]; star). Bottom panel: The distribution of the completeness of the between-pathway synthetic lethal interactions in our identified pathway pairs.

Our search generated 2,590 pathway pairs (7.7±3.1 genes per pair), which cover 5,284 (56% of 9,376) synthetic lethal interaction pairs involving 689 yeast genes. Since we did not require the physical interaction to support our pathway models, it was expected that the pathways we identified to be larger than that identified by integrating genetic and physical interaction data [1], [8]. To test this hypothesis, we compared the sizes of the pathways from different methods. As shown in Figure 3 upper panel, the pathways (redundancy removed, see Methods) identified by our method were significantly (P<1e-16, Wilcox rank sum test) larger than that of Kelley and Ideker [1], whereas the sizes of the pathways in Kelley and Ideker [1] was significantly (P<1e-6, Wilcox rank sum test) larger than that of Ulitsky and Shamir [8]. In addition, our investigation suggested that this result is not an artifact of parameters specific to our method (Figure S1 and Text S1). A close inspection revealed that many pathways (40%; 110/280) identified by Ulitsky and Shamir [8] had one pathway member of size 2 (however, we also noted that Ulitsky and Shamir [8] had identified 5 pathways with more than 50 genes and the largest one had 201 genes). This result implied that we can potentially increase the size of gene sets at the cost of losing the physical interaction support. However, the pathways we identified can sometimes be smaller than the congruence score method by Ye et al [7]. For example, BIK1 was absent from the dynein-dynactin checkpoint pathway, as shown in Figure 2A, and it was identified by the congruence score method. It should be noted that pathway size distribution is not a measure of accuracy of our method. In fact, the size of a pathway could be very arbitrary if we take a gene-network perspective, where a pathway is used to highlight a functionally homogenous sub-network (see section “Pathway definition and identification”). However, we noted that in functional genomics a practical problem is that many genes are not functionally annotated, thus it may be helpful to provide biologists reasonably larger lists of genes showing compensatory interaction patterns. In fact, the example in Figure 2C discussed previously suggests the value of providing a larger pathway.

We next asked how the synthetic lethal interactions were depleted from either pathway of each pathway pair and how the synthetic lethal interactions were enriched between pathways in each pair. Since we imposed a stringent constraint on the within-pathway synthetic lethal interactions (α = 0.01; see Method), we found that only one of the pathways we identified have within-pathway synthetic lethal interactions. Thus we proceeded to determine how the synthetic lethal interactions were enriched between pathways (defined as completeness *d_12_*, which represents the proportion of all potential synthetic lethal interactions that have been observed; see Equation (1)). According to the assumptions in Figure 1A, if all possible pairs of genes between the two compensatory pathways are connected by synthetic lethal interactions, they have a completeness of 100%. In practice, two compensatory pathways may have a completeness less than 100%, due to either lack of experiments or biological reasons. As can be seen from Figure 3 bottom panel, the pathway pairs we identified had a typical completeness of between-pathway synthetic lethal interactions at the range of [0.75, 0.85], which may have indication to the false-negative rate of the synthetic lethal interaction data (Text S2). Noticeably, some pathway pairs we identified had completeness *d_12_* = 100%.

We also determined the statistical significance of the pathway pairs we identified. To achieve this goal, we generated 10,000 random networks by crossing pairs of edges as done in Kelley and Ideker [1] and Milo et al [19]. For each pathway pair we identified (with completeness *d_1_*, *d_2_* and *d_12_*; see Equation (1)), we counted the chances of observing this pathway pair to have *d*_1_^0^≤*d*_1_, *d*_2_^0^≤*d*_2_ and *d*_12_^0^≥*d*_12_, where the superscript 0 means the score was under permutation. As it turned out, all pathway pairs we identified had P-value less than 1e-4. This result suggests that the constraint β = 0.75 of Equation (1) was rather strict. In fact, we found from our permutation study that given *d*_1_^0^≤0.01and *d*_2_^0^≤0.01, *d*_12_^0^ had a mean of 0.09 and a standard deviation of 0.06 (Figure S2 and Text S2), which further suggests that our threshold β = 0.75 of Equation (1) was very strict.

### Physical interactions in the discovered pathways

**Figure 4:**
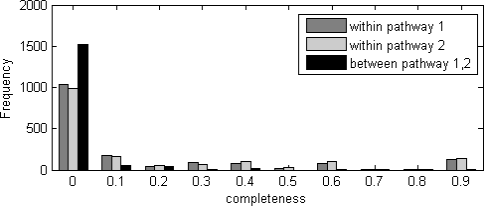
Physical interactions are enriched in pathways we identified and depleted from between-pathway gene pairs.

Biologically, we expected the enrichment of physical interactions within pathways we identified, which was an important component in Kelley and Ideker [1] and Ulitsky and Shamir [8]. To study the enrichment of physical interactions within the pathways identified by our method, we downloaded the physical interaction data collected by Ulitsky and Shamir [8], which included 67,856 physical interaction pairs. For each pathway pair identified, we calculated the completeness scores *d_1_*, *d_2_*, *d_12_* as defined in Equation (1) using the physical interaction data to measure the enrichment of physical interactions within pathway 1, within pathway 2, and between pathway 1 and pathway 2, respectively. Similar to the idea of Kelly and Ideker [1], we expected completeness scores *d_1_* and *d_2_* to be large. On the other hand, we hypothesized that physical interactions will be less enriched between compensatory pathway pairs, i.e., *d_12_* to be relatively small, according to Figure 1A. The distribution of completeness was shown in Figure 4. As can be seen, there were significantly (P-value<10^−16^, Wilcox rank sum test, one-sided) more within-pathway physical interactions than between-pathway physical interactions, suggesting that our identified pathway pairs do have biological meanings.

### Functional homogeneity of discovered pathways

**Figure 5:**
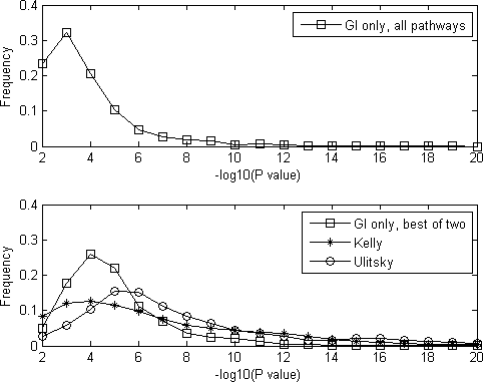
The distribution of the number of identified pathways as a function of the functional homogeneity. Upper panel: the functional homogeneity of all the pathways from each pathway pair identified by our method. Although a considerable percentage of pathways identified by our method had high homogeneity scores, many of them showed a low functional homogeneity due to multifunction effects. Bottom panel: at least one of the two pathways of each pathway pair identified by our method had high homogeneity. Note that the functional homogeneity score was still significantly lower than that of the pathways identified using physical interaction data.

We next sought to study how different cellular functions were enriched in the pathways we identified. The functional annotation from Gene Ontology [12] was used for the evaluation. For each pathway we identified, a hypergeometric distribution was used to test the enrichment of genes annotated with a specific Gene Ontology concept. Since there were 1720 Gene Ontology biological process concepts being screened (see Methods), the −log10 of the smallest P-value of all the 1720 P-values (not corrected for multiple testing) was defined as the functional homogeneity score of our pathways. Thus, a higher homogeneity score implied a better grouping of genes into pathways. To provide a comparison, we also ran the same enrichment test on the pathways (redundancy removed, see Methods) identified by Kelley and Ideker [1] and Ulitsky and Shamir [8]. As expected, the pathways identified by our method (Figure 5, upper panel) had lower functional homogeneity than those identified with the aid of physical interaction data (Figure 5, bottom panel, stars and circles).

However, an interesting observation was that in each pathway pair identified by our method, at least one of the two pathways had high functional homogeneity. To show this fact, we took the pathway with a smaller P-value from each pathway pair identified by our method and drew the distribution of their functional homogeneity (redundancy removed, see Methods). As shown in Figure 5 bottom panel (boxes), these pathways showed enhanced functional homogeneity compared to Figure 5 upper panel (although still lower than that identified using physical interaction support; Figure 5, bottom panel, stars and circles). This phenomenon was actually a result of the multifunction effects of the pathways. To explain, suppose a pathway A has compensatory effects with pathways B and C. Then with a high probability our method will group genes from pathway A into one set and genes from pathways B and C into another set, resulting in one genuine pathway corresponding to A with a high functional homogeneity score and another spurious pathway corresponding to B and C, with a low functional homogeneity score. Thus, at the cost of reducing the accuracy of one of the two pathways in each identified pathway pair, it was still possible to gain much insight of the gene functions using synthetic lethal interaction data alone.

### Network of compensatory biological functions

**Figure 6:**
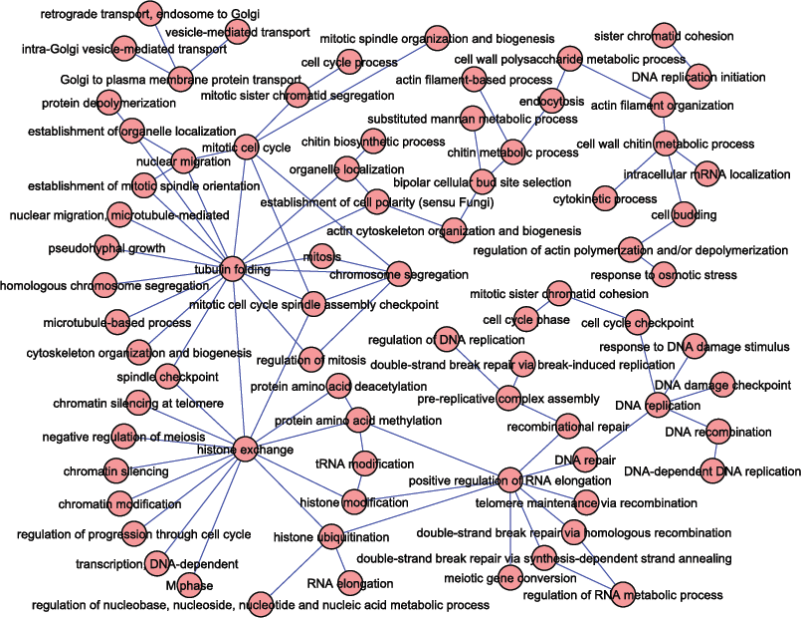
Network of compensatory biological functions revealed by synthetic lethal interaction data. Each node represents a Gene Ontology concept. Each edge represents the existence of extensive synthetic lethal interactions between gene-pairs from the two connected Gene Ontology concept pairs.

The above result showed that our method can group genes into pathway pairs, and in most cases at least one pathway from each pathway pair had specific biological functions. Since synthetic lethal interaction data often predict functionally compensatory pathways [1], [7]–[8], we next determined how different cellular functions compensated each other. To make the analysis strict, we selected the pathways pairs where each member pathway had a functional homogeneity score larger than 5.24 (or P-value 5.8e-6, which corresponds to the bonferroni-corrected P-value 0.01) and more than 30% of the member genes in each pathway had the same function as the one annotated to the pathway. This requirement resulted in 89 non-redundant pairs of Gene Ontology concepts connected by extensive synthetic lethal interactions (Table S1). As shown in Figure 6, the Gene Ontology concepts could be arranged in a network. For example, the genes involved in *Golgi to plasma membrane protein transport* were found to be synthetic lethal with the genes involved in *intra-Golgi vesicle-mediated transport*; *retrograde transport, endosome to Golgi*; *and vesicle-mediated transport*.

Interestingly, the obtained network showed considerable un-evenness toward several Gene Ontology concepts, such as *tubulin folding* and *histone exchange*. For example, the *histone exchange* was found to be connected to many other functions, such as *transcription*, *DNA dependent*; *negative regulation of meiosis*; *chromatin silencing*; *tubulin folding*; etc. Another hub node was the *positive regulation of RNA elongation*, which was also connected to many functions, mostly involving *DNA repair* biology. The underlying biological implications await additional exploration.

### Multifunction effects of pathways

Since we deliberately excluded the physical interaction data when searching for compensatory pathway pairs (see Methods), it was not surprising that sometimes we discovered pathways containing members that obviously deviated from the most common function of these pathways (Figure 2). At least three possibilities can explain this phenomenon. First, false positives might exist in the synthetic lethal interaction data set. Thus, an obviously unrelated gene may be incorporated into one pathway. However, the current synthetic lethal interaction data is believed to contain only few false positives [5]. Thus this explanation is unlikely true. Second, the “strange member” might be a new member of that cellular function. In other words, the Gene Ontology data contains false positives and false negatives. The existence of physical interactions between this new gene and other genes known to belong to that function will increase the probability of this explanation. Third, it might be simply due to multifunction (Figure 1C). In this case, the multiple functions contained in one identified pathway may suggest that its collaborator pathway participates in more than one cellular function.

The third consideration was identified in this analysis. In Figure 2E, the prefoldin complex (GIM3, GIM4, GIM5, YKE2, PAC10) was found to be compensatory to the spindle checkpoint complex (BUB1, BUB2, BUB3, MAD1, MAD2, BMH1). However, the VID21 gene was found to be grouped with prefoldin complex, which had synthetic lethal interaction with spindle checkpoint complex except BMH1. VID21 is a component of the NuA4 histone acetyltransferase complex. Thus, this fact suggested that the spindle checkpoint complex may also participate in a function in collaboration with histone modification. In this sense, we determined that the spindle check point was a multifunction pathway.

The multifunction effect of pathways is better illustrated using a network representation in Figure 6. The fact that a pathway with a given function may be connected to many other pathways, each with a distinct function, suggests the multifunction effects of a pathway. In this sense, the “hub” pathways such as those involved in positive regulation of RNA elongation and histone exchange are good examples of pathways with multifunction effects.

## Discussion

High throughput bio-techniques are generating more and more extensive descriptions of the gene networks. Unlike physical interaction data, which is conceptually straightforward, the synthetic lethal interaction data implies structural properties of the gene network at a higher level. Namely, synthetic lethal interactions often imply compensatory pathway structures, while physical interactions suggest that the participating genes reside in the same pathway. Thus, integrating synthetic lethal interaction and physical interaction data is an efficient way to gain biological insights from the network data. In particular, enrichment of physical interactions such as protein-protein and protein-DNA interactions within a set of genes increases our confidence that the gene set is biologically meaningful. For example, the work of Kelley and Ideker [1] and Ulitsky and Shamir [8] revealed a great number of pathways supported by physical interactions. However, the size of the pathways identified by them could be very small, sometimes with only two genes. We postulate the reason is that the current physical interaction data is far from complete and therefore it will be difficult to reconstruct most pathways. Although the size distribution of pathways is still an unknown fact, it is likely that many pathways consist of more than two genes. In fact, our example in Figure 2C demonstrates that the small pathway size can be a consequence of lacking physical interaction support. We stress that in functional genomics a practical problem is that many genes are not functionally annotated, thus it may be helpful to provide biologists larger list of candidate genes showing compensatory interaction patterns. More importantly, given that in some model organisms only genetic screening data is available, our findings suggest that we can obtain substantial biological insights about genetically redundant pathways without inquiring the physical interaction data.

The mannan/transport compensation of chitin synthesis identified here exemplifies some important features that should be considered when using genetic interactions alone to predict compensatory pathways. One potential weakness in predicting compensatory interactions is that there is a minor subset of physically interacting genes that are also synthetically lethal. In the analysis presented here HOC1 was identified as a chitin synthase member, likely due to within-pathway redundancy, while MNN10 and VAN1 were correctly identified as mannan synthesis genes as a result of their genetic interactions with chitin synthesis genes. However, published data on mannan synthesis [14] allowed for a quick interpretation of the HOC1 interaction with VAN1 as a probable within-function interaction.

Another feature of this analysis is that one pathway is often compensated by multiple pathways, as exemplified by cytoskeletal components (necessary for moving complexes through the cell) and the two mannan polymerase complexes that were both identified as belonging to the same group of proteins compensating chitin synthesis. Ulitsky and Shamir identified a CHS3/BIN4/SKT5 complex compensation by both MY02/SHE4 and VRP1/SLA1 separately (BMP-48 and -130, respectively; [8]). In predicting a larger set of interactions, this analysis yielded a more complete picture of chitin compensating functions than previous analyses with these data that included physical interactions. A result of such fragmentation presents the possibility that pieces of the whole interaction will be missed due to human error and indicates the requirement of more post analysis evaluation of the data to achieve the same big-picture view provided with the approach presented here. Given that there are also errors in the protein interaction data that must be considered when vetting the output of analyses based on genetic and protein interactions, the production of a larger list of interactions in one cluster is likely to save researchers time and error at the end of analysis.

Additionally, from a biological perspective, it is one thing to singularly know either that chitin synthesis mutations can be compensated by a mannan polymerase or by a few secretion polarity molecules; and a completely different thing to see that there are several secretion polarity mechanisms and two mannan polymerase complexes that interact with chitin synthesis mutants. A partitioned view of the interactions among groups of genes (or a big-picture created from a poorly reconstructed set of smaller interactions) limits the types of questions that can be asked of the analyses (i.e. “How many cellular systems affect chitin synthesis?”), whereas, simpler questions (such as “Does mannan synthesis compliment chitin synthesis?”) are unaffected by having a fuller picture of how genes interact, as subsets of the output can be ignored. Moreover, the interactions provided by assessing the data used with this method have been demonstrated to a much greater degree in subsequent analyses of chitin synthesis synthetic lethal analyses [16], indicating that the expanded list of interactions was also more appropriate in this case.

Several parameters in this analysis require optimization. It is necessary to determine optimal values for pathway size, completeness, and how the sizes of each pathway in a compensatory pathway pair are balanced. However, several practical considerations make this study challenging. First, the current synthetic lethal interaction network is heavily biased. Due to the formidable number of possible combinations (∼6000×6000/2) and the sparse nature of synthetic lethal interactions [5], the query genes are generally selected carefully, despite the fact that all genes are used as bait. Thus, any study of the global characteristics of the gene network should take special care with this sampling bias. Second, the evaluation standard of pathways, such as the Gene Ontology, is a fast evolving standard. Other well studied metabolic pathways, such as that documented in the KEGG database [20], have few, if any, overlap with the current query genes. The lack of an extensive definition of pathways focusing on the current query genes makes it hard to accurately evaluate parameter estimations.

In addition to the binary synthetic lethal interactions, quantitative genetic epistasis data [21] can be another rich source of genetic interactions. Epistasis refers to the phenomenon in which the phenotypic consequence of altering one gene is differentially modulated by the specific alleles of a second one, including both negative (aggravating) interactions and buffering (relieving) interactions [21]. Synthetic lethal interaction is an extreme of the negative interactions. It has been demonstrated that the quantitative epistasis data contains rich information regarding pathways and protein complexes [21]. More importantly, due to the existence of essential genes [22], analyses focusing on synthetic lethal interactions are not fully genome-wide and thus not really systematic. With the advance of biotechnology, such as *d*ecreased *a*bundance by *m*RNA *p*erturbation (DAmP) [21] and promoter-replacement techniques [23], genetic interaction data are being generated in a systematic fashion. Future work will be devoted to studying this broader network of interactions.

## Materials and Methods

### Data

We downloaded the synthetic lethal interaction data from BIOGRID ([6]; version 2.0.31). It contains 9376 non-redundant synthetic lethal genetic interactions, involving 2348 yeast genes. Note that in this paper we focused on the synthetic lethal genetic interactions. Other genetic interactions, such as 7,233 synthetic growth defect and synthetic rescue interactions were not included.

The 68172 protein-protein and protein-DNA interaction data covering 6814 yeast genes was obtained from Ulitsky and Shamir [8]. The functional annotation data of yeast genes is downloaded from SGD [24], as of August 2007. We parsed the Gene Ontology [12] data structure and mapped yeast genes to all ontology nodes and the resulting 1720 biological process nodes, each with no more than 500 genes, are selected to perform functional analysis.

### Network visualization

Network figures were created using *Cytoscape* [25].

### Algorithm

By viewing genes as nodes and synthetic lethal interactions between genes as edges, the synthetic lethal interaction data can be represented as a network. Our goal was to identify approximately complete bipartite graphs within the synthetic lethal interaction network which satisfied the following criteria: 1) there were no or few edges within each sub-network; 2) there was an abundance of edges connecting the sub-network pairs; 3) the sub-networks contained at least four genes. Mathematically, we denote the original network as *G*(*V*, *E*), where *V* is the set of yeast genes and *E* is the set of edges connecting yeast genes: each element *e_ij_* in *E* takes value 1 if there is synthetic lethal interaction between genes *i* and *j* and 0 otherwise. Our goal was to find all node set pairs (*V1*, *V2*) that satisfied
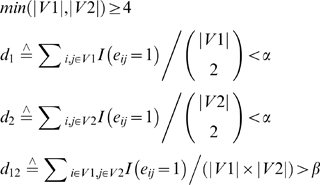
(1)where *I*(•) is the identity function, |•| is a function on sets, counting the number of elements in it. α and β are any pre-chosen numbers so that α is close to 0 and β is close to 1. Here we set α = 0.01, β = 0.75 (Text S3 and Text S2).

Another consideration was the balance between the sizes of node set pair (*V1*, *V2*). Due to the formidability of the searching space, we wanted to first focus on the sub-network pairs with similar sizes. Taking the above issues into consideration, we use the following objective function to search for candidate node set pairs:
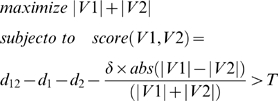
(2)where δ is a tuning parameter to control the penalty to the size differences between node sets *V1* and *V2* and *abs*(•) is the absolute value. A larger δ gives more penalties to the size differences between set *V1* and *V2*. Here we set δ = 1.5 (Figure S3, Figure S4 and Text S4).

To implement the above objective, we proposed the following heuristic algorithm.

**Input**: (1) *G*(*V*, *E*), the network;
  (2) α = 0.01, β = 0.75, *T* = 0.4, parameters in Equation (2).
**Output**: *R* = (*G_1_*, *G_2_*, …, *G_m_*), superset of *m* sub-networks *G_i_*(*V1*,*V2*,*E′*) to be discovered and returned.

1: *R* = Φ. *C* = 0.

2: **repeat**

3:  Set *V1* = Φ, *V2* = Φ. *S*(*V1*, *V2*, *E′*) is the sub-network pair we will discover.

4:  Randomly select an edge *e_ij_* = 1 from *E*. Set *V1* = {*i*}, *V2* = {*j*}.

5:  **while** there are nodes that can be added to *S* **do**

6:   Find the set of all nodes in *G* that are connected to *S*: *V*′ = {*v* | *v*∈*V* and *e_vk_* = 1, for some *k*∈*V*1∪*V*2}

7:   **if** *V′* is empty **then**

8:    **break**

9:   **for** all *v*∈*V*′ **do**

10:    Calculate *score*(*v*) = max(*score*(*V*1∪*v*, *V*2), *score*(*V*1, *V*2∪*v*))

11:    set *score*(*v*) = 0 if *score*(*v*)<*T*

12:   **end for**

13:   **if** score(v) = 0 for all *v*∈*V*′

    **break**

14:   sample a node *v* randomly from *V′* according to vector *score*(*v*), *v*∈*V*′

15:   add *v* to *V1* if *score*(*V*1∪*v*, *V*2)> = *score*(*V*1, *V*2∪*v*); to *V2* otherwise

16:  **end while**

17:  add *S* into *R* if |*V1*|>3, |*V2*|>3, and *S*∉*R*

18:  *C* = *C*+1

19: **until** *C*>*C* ^*^

Our algorithm randomly chose an edge from the whole set of edges and initialized the sub-network pair *V1* and *V2*. It then enumerated all other nodes and assigned them score according to Equation (2). We then sampled a node from these candidates according to the probability proportional to their scores. Note that a candidate with a score less than our threshold (*T* = 0.4) was not sampled. The sampled node was added into the sub-network according to its score. A pre-defined number C* ( = 10000 in this work) of searches are performed.

### Redundancy

Similar to Kelley and Ideker [1], repeat pathway pairs were removed. Specifically, if pathway pair A and pathway pair B shared more than 50% synthetic lethal interaction edges, the smaller pathway pair was removed. For summary statistics and function enrichment analysis of the identified pathways, if pathway A and B shared more than 50% member genes, the smaller pathway was removed. The same procedure applies to pathways from Kelley and Ideker [1] and Ulitsky and Shamir [8].

## Supporting Information

Figure S1 Comparison of the size distribution of pathways identified by our method with that identified by Kelly/Ideker and Ulitsky/Shamir, using the same size constraint parameters and original datasets. Upper panel: size distribution of non-redundant pathways identified by our algorithm on the data used by Kelly/Ideker; Bottom panel: size distribution of non-redundant pathways identified by our algorithm on the data used by Ulitsky/Shamir.
(0.02 MB TIF)

Figure S2 Permutation distribution of β. A large fraction (21%) of permutation β had value of 0. For non-zero β, the mean was 0.091 and the standard deviation was 0.058.
(0.01 MB TIF)

Figure S3 Distribution of pathway size differences under different penalty parameter δ.
(0.01 MB TIF)

Figure S4 Distribution of pathway completeness under different penalty parameter δ.
(0.01 MB TIF)

Text S1 Minimum pathway size parameter
(0.03 MB DOC)

Text S2 Between-pathway completeness β.
(0.03 MB DOC)

Text S3 Within-pathway completeness α.
(0.02 MB DOC)

Text S4 Pathway size imbalance penalty δ.
(0.03 MB DOC)

Table S1 Network of compensatory biological functions.
(0.05 MB XLS)

## References

[1] Kelley R, Ideker T (2005). Systematic interpretation of genetic interactions using protein networks.. Nat Biotechnol.

[2] Boone C, Bussey H, Andrews B (2007). Exploring genetic interactions and networks with yeast.. Nat Rev Genet.

[3] Giaever G, Chu A, Ni L, Connelly C, Riles L (2002). Functional profiling of the Saccharomyces cerevisiae genome.. Nature.

[4] Tong A, Evangelista M, Parsons A, Xu H, Bader G (2001). Systematic genetic analysis with ordered arrays of yeast deletion mutants.. Science.

[5] Tong A, Lesage G, Bader G, Ding H, Xu H (2004). Global mapping of the yeast genetic interaction network.. Science.

[6] Stark C, Breitkreutz B, Reguly T, Boucher L, Breitkreutz A (2006). BioGRID: a general repository for interaction datasets.. Nucleic Acids Res.

[7] Ye P, Peyser B, Pan X, Boeke J, Spencer F (2005). Gene function prediction from congruent synthetic lethal interactions in yeast.. Mol Syst Biol.

[8] Ulitsky I, Shamir R (2007). Pathway redundancy and protein essentiality revealed in the Saccharomyces cerevisiae interaction networks.. Mol Syst Biol.

[9] Uetz P, Giot L, Cagney G, Mansfield T, Judson R (2000). A comprehensive analysis of protein-protein interactions in Saccharomyces cerevisiae.. Nature.

[10] Ito T, Tashiro K, Muta S, Ozawa R, Chiba T (2000). Toward a protein-protein interaction map of the budding yeast: A comprehensive system to examine two-hybrid interactions in all possible combinations between the yeast proteins.. Proc Natl Acad Sci U S A.

[11] Harbison C, Gordon D, Lee T, Rinaldi N, Macisaac K (2004). Transcriptional regulatory code of a eukaryotic genome.. Nature.

[12] Ashburner M, Ball C, Blake J, Botstein D, Butler H (2000). Gene ontology: tool for the unification of biology. The Gene Ontology Consortium.. Nat Genet.

[13] Karp G (1999). Cytoplasmic membrane systems: structure, function, and membrane trafficking.. Cell and Molecular Biology: Concepts and Experiments. 2nd edition.

[14] Munro S (2001). What can yeast tell us about N-linked glycosylation in the Golgi apparatus?. FEBS Lett.

[15] Yoko-o T, Wiggins C, Stolz J, Peak-Chew S, Munro S (2003). An N-acetylglucosaminyltransferase of the Golgi apparatus of the yeast Saccharomyces cerevisiae that can modify N-linked glycans.. Glycobiology.

[16] Lesage G, Shapiro J, Specht C, Sdicu A, Ménard P (2005). An interactional network of genes involved in chitin synthesis in Saccharomyces cerevisiae.. BMC Genet.

[17] Wiggins C, Munro S (1998). Activity of the yeast MNN1 alpha-1,3-mannosyltransferase requires a motif conserved in many other families of glycosyltransferases.. Proc Natl Acad Sci U S A.

[18] Osmond BC, Specht CA, Robbins PW (1999). Chitin synthase III: synthetic lethal mutants and “stress related” chitin synthesis that bypasses the CSD3/CHS6 localization pathway.. Proc Natl Acad Sci U S A.

[19] Milo R, Shen-Orr S, Itzkovitz S, Kashtan N, Chklovskii D (2002). Network motifs: simple building blocks of complex networks.. Science.

[20] Kanehisa M, Goto S, Hattori M, Aoki-Kinoshita K, Itoh M (2006). From genomics to chemical genomics: new developments in KEGG.. Nucleic Acids Res.

[21] Schuldiner M, Collins S, Thompson N, Denic V, Bhamidipati A (2005). Exploration of the function and organization of the yeast early secretory pathway through an epistatic miniarray profile.. Cell.

[22] Winzeler EA, Shoemaker DD, Astromoff A, Liang H, Anderson K (1999). Functional characterization of the S. cerevisiae genome by gene deletion and parallel analysis.. Science.

[23] Davierwala AP, Haynes J, Li Z, Brost RL, Robinson MD (2005). The synthetic genetic interaction spectrum of essential genes.. Nat Genet.

[24] Nash R, Weng S, Hitz B, Balakrishnan R, Christie K (2007). Expanded protein information at SGD: new pages and proteome browser.. Nucleic Acids Res.

[25] Shannon P, Markiel A, Ozier O, Baliga N, Wang J (2003). Cytoscape: a software environment for integrated models of biomolecular interaction networks.. Genome Res.

