# Supplementary figures and images for: Mapping Genetically Compensatory Pathways from Synthetic Lethal Interactions in Yeast
Source: PLoS One. 2008 Apr 9;3(4):e1922. doi: 10.1371/journal.pone.0001922 (PMC2275788; doi:10.1371/journal.pone.0001922)

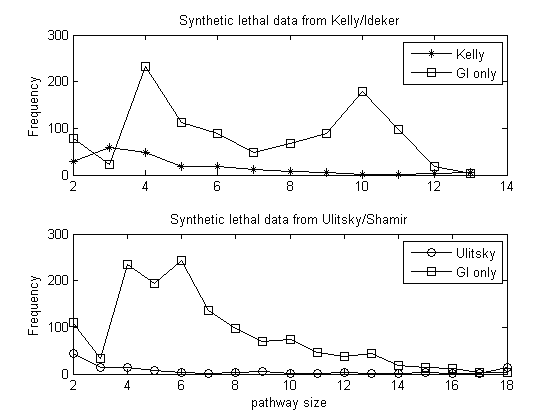

Supplement: Figure S1 — Comparison of the size distribution of pathways identified by our method with that identified by Kelly/Ideker and Ulitsky/Shamir, using the same size constraint parameters and original datasets. Upper panel: size distribution of non-redundant pathways identified by our algorithm on the data used by Kelly/Ideker; Bottom panel: size distribution of non-redundant pathways identified by our algorithm on the data used by Ulitsky/Shamir. (0.02 MB TIF) [file pone.0001922.s001.tif]

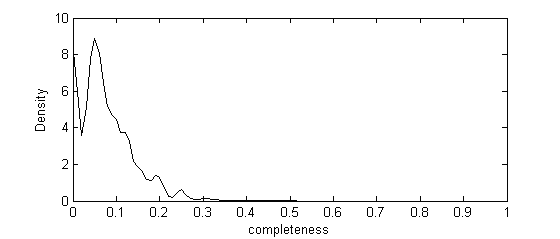

Supplement: Figure S2 — Permutation distribution of β. A large fraction (21%) of permutation β had value of 0. For non-zero β, the mean was 0.091 and the standard deviation was 0.058. (0.01 MB TIF) [file pone.0001922.s002.tif]

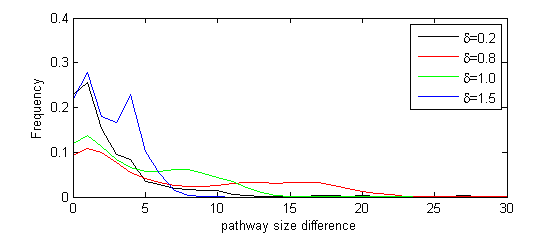

Supplement: Figure S3 — Distribution of pathway size differences under different penalty parameter δ. (0.01 MB TIF) [file pone.0001922.s003.tif]

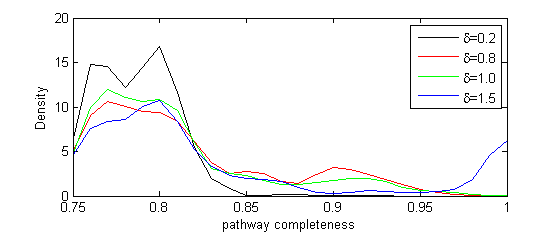

Supplement: Figure S4 — Distribution of pathway completeness under different penalty parameter δ. (0.01 MB TIF) [file pone.0001922.s004.tif]
