# Supplementary material for: Mapping Genetically Compensatory Pathways from Synthetic Lethal Interactions in Yeast
Source: PLoS One. 2008 Apr 9;3(4):e1922. doi: 10.1371/journal.pone.0001922 (PMC2275788; doi:10.1371/journal.pone.0001922)
Supplement: Text S1 — Minimum pathway size parameter (0.03 MB DOC) [file pone.0001922.s005.doc]

Text S1

Effect of parameters on identifying pathways

**Minimum pathway size parameter**

In Figure 3 upper panel, we compared the size distribution of our final identified pathways with that identified by Kelly and Ideker [1] and Ulitsky and Shamir [2]. However, it should be noted that we used a constraints of at least 4 genes in each member pathway of a given compensatory pathway pair. Thus, the comparison is unfair. To make a fair comparison, we studied the effect of the minimum pathway size parameter. As can be seen from Figure S1, the pathways identified by our algorithm generally have a larger size compared to that identified by Kelly and Ideker [1] and Ulitsky and Shamir [2], where the comparison is based on the genetic interaction data sets and size parameters used by the original authors, respectively. As can be seen, the parameter of pathway size did not dramatically affect our conclusion that our algorithm can return bigger pathways.

It is interesting to note that using Kelly/Ideker data, the pathways identified by our method have two modes of pathway sizes: 4 and 10, which is more similar to our result in Figure 3 upper panel than that using Ulitsky/Shamir data. The reason is that in this paper we used synthetic lethal data only, which increased from 4812 in Kelly and Ideker [1] to 9376 in our current database. On the other hand, Ulitsky/Shamir data contains 12850 genetic interaction pairs, where synthetic fitness interaction data is included [2]. The synthetic fitness interaction data, which we considered to be in the class of quantitative genetic interactions (section “Discussion” of main text), may not be as accurate as synthetic lethal interaction data. Thus it could dramatically shift the size distribution of pathways identified by other method. In fact, the pathways identified by Ulitsky and Shamir [2] are generally smaller than that identified by Kelly and Ideker [1], as can be seen from Figure S1 and Figure 3 upper panel in main text.

References

1. Kelley R, Ideker T (2005) Systematic interpretation of genetic interactions using protein networks. Nat Biotechnol 23: 561-566.

2. Ulitsky I, Shamir R (2007) Pathway redundancy and protein essentiality revealed in the Saccharomyces cerevisiae interaction networks. Mol Syst Biol 3: 104.
