# Supplementary material for: Mapping Genetically Compensatory Pathways from Synthetic Lethal Interactions in Yeast
Source: PLoS One. 2008 Apr 9;3(4):e1922. doi: 10.1371/journal.pone.0001922 (PMC2275788; doi:10.1371/journal.pone.0001922)
Supplement: Text S2 — Between-pathway completeness β. (0.03 MB DOC) [file pone.0001922.s006.doc]

Text S2

Effect of parameters on identifying pathways

**Between-pathway completeness β**: In the general understanding of redundant pathways, between-pathway synthetic lethal interaction patterns were expected to be enhanced (text of Figure 1A in Ye et al. [1]). To obtain a null distribution of β where no genetically redundant pathway pairs exist, we generated many random networks by randomly crossing edges within the whole synthetic lethal interaction network. We studied how the pathways pairs we identified in this paper behaved within those random networks. To achieve this goal, we calculated their β values when their α values satisfied the within-pathway completeness criteria, i.e., less than 0.01. As shown in Figure S2, the null distribution of β centered around 0.09 with a standard deviation 0.058, suggesting that our threshold β=0.75 was very strict. We also noted that in Tong et al. [2] the estimated false negative rate was 17~41% in synthetic lethal interaction screening experiments. Thus the “missed” (incomplete) synthetic lethal interactions in our pathway pairs may correspond to those false negatives. We set β=0.75 to take this fact into consideration.

References

1. Ye P, Peyser B, Pan X, Boeke J, Spencer F, et al. (2005) Gene function prediction from congruent synthetic lethal interactions in yeast. Mol Syst Biol 1: 2005.0026.

2. Tong A, Lesage G, Bader G, Ding H, Xu H, et al. (2004) Global mapping of the yeast genetic interaction network. Science 303: 808-813.
