# Supplementary material for: Mapping Genetically Compensatory Pathways from Synthetic Lethal Interactions in Yeast
Source: PLoS One. 2008 Apr 9;3(4):e1922. doi: 10.1371/journal.pone.0001922 (PMC2275788; doi:10.1371/journal.pone.0001922)
Supplement: Text S3 — Within-pathway completeness α. (0.02 MB DOC) [file pone.0001922.s007.doc]

Text S3

Effect of parameters on identifying pathways

**Within-pathway completeness α**: As illustrated in Figure 1A of Ye et al. [1], synthetic lethal interactions should be reduced in the “same functional pathway” of either pathway of a genetically redundant pathway pair. In this paper we referred to this fact as reduced “within-pathway completeness”. The depletion of synthetic lethal interactions was also observed from the results in Kelley and Ideker [2] and Ulitsky and Shamir [3]. Thus, in this paper we set within-pathway completeness α=0.01. This value is higher than the false positive rate in synthetic lethal interaction screening, which is believed to be very low. The rationale for this choice was that we not only wanted to accommodate within-pathway synthetic lethal interactions, which are in fact false positives, but also wanted to accommodate a few within-pathway synthetic lethal interactions which exist for some unknown reasons. A simple calculation indicated that there could be a within-pathway interaction only when the pathway had more than 15 genes ($0.01\times15\times14/2=1.05$). We found only one pathway pair having this property.

References

1. Ye P, Peyser B, Pan X, Boeke J, Spencer F, et al. (2005) Gene function prediction from congruent synthetic lethal interactions in yeast. Mol Syst Biol 1: 2005.0026.

2. Kelley R, Ideker T (2005) Systematic interpretation of genetic interactions using protein networks. Nat Biotechnol 23: 561-566.

3. Ulitsky I, Shamir R (2007) Pathway redundancy and protein essentiality revealed in the Saccharomyces cerevisiae interaction networks. Mol Syst Biol 3: 104.
