# Supplementary material for: Mapping Genetically Compensatory Pathways from Synthetic Lethal Interactions in Yeast
Source: PLoS One. 2008 Apr 9;3(4):e1922. doi: 10.1371/journal.pone.0001922 (PMC2275788; doi:10.1371/journal.pone.0001922)
Supplement: Text S4 — Pathway size imbalance penalty δ. (0.03 MB DOC) [file pone.0001922.s008.doc]

Text S4

Effect of parameters on identifying pathways

**Pathway size imbalance penalty δ**: In this paper we artificially gave penalties to the score of pathway pairs with different sizes: with a higher penalty for a greater disparity. We thus sought to study how δ affected the size difference of the pathway pairs we identified. As shown in Figure S3, the effect of δ was clear: larger δ tended to yield pathway pairs of similar sizes; while smaller δ tended to yield pathway pairs of different sizes. However, it should be noted that our introduction of δ was for a technical reason: the result space was too large and we wanted to first focus on pathways of similar sizes. It is possible that removing this constraint would be a better choice, which will be a subject of future study.

We next sought to study how δ affected the completeness of the pathway pairs we identified. As shown in Figure S4, the completeness value was robust to different δ values. This result suggests that our technical introduction of δ did not affect the quality of the discoveries.
